# Proteomics of rice grain under high temperature stress

**DOI:** 10.3389/fpls.2013.00036

**Published:** 2013-03-06

**Authors:** Toshiaki Mitsui, Takeshi Shiraya, Kentaro Kaneko, Kaede Wada

**Affiliations:** ^1^Department of Applied Biological Chemistry, Faculty of Agriculture, Niigata UniversityNiigata, Japan; ^2^Graduate School of Science and Technology, Niigata UniversityNiigata, Japan

**Keywords:** α-amylase, chalky grain, high temperature stress, *Oryza sativa*, ripening, starch

## Abstract

Recent proteomic analyses revealed dynamic changes of metabolisms during rice grain development. Interestingly, proteins involved in glycolysis, citric acid cycle, lipid metabolism, and proteolysis were accumulated at higher levels in mature grain than those of developing stages. High temperature (HT) stress in rice ripening period causes damaged (chalky) grains which have loosely packed round shape starch granules. The HT stress response on protein expression is complicated, and the molecular mechanism of the chalking of grain is obscure yet. Here, the current state on the proteomics research of rice grain grown under HT stress is briefly overviewed.

## INTRODUCTION

**FIGURE 1:**
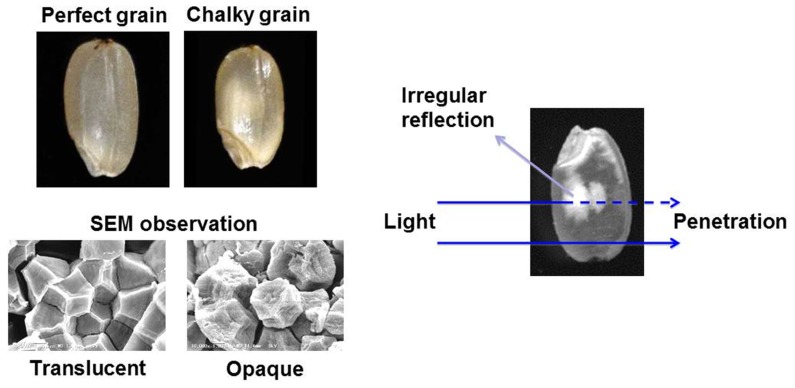
**Perfect and chalky grains of rice.** Bars: 3.33 μm.

The Intergovernmental Panel on Climate Change is discussing several scenarios concerning the greenhouse gas emission, and it is predicted that the global surface temperature will further increase during the twenty-first century. High temperature (HT) impediment in developing stage of crops, such as rice (*Oryza sativa*), maize (*Zea mays*), wheat (*Triticum* spp.), barley (*Hordeum vulgare*) and soybean (*Glycine max*), has been occurred due to the impact of global warming (Ainsworth and Ort, 2010). Rice production is known to be sensitive to increasing environmental temperature (Peng et al., 2004), and current grain filling temperatures are already approaching critical levels in many countries with rice cultivation (Ainsworth, 2008). Furthermore, it should be stressed that the grain quality is more susceptible to the HT stress compared with the grain yield. The appearance quality of rice grain is mainly evaluated by its transparency. As shown in **Figure 1**, the perfect grain is filled with normal starch granules exhibited polygonal with sharp edges. In the case of damaged (central chalky) grains caused by the HT stress, abnormal and round shape starch granules were loosely packed in the part of grain, and this part is whitely seen by irregular reflection of the light. The mechanism of grain chalkiness under HT stress is considerably complicated. The temperature at the grain filling stage has shown to influence the starch composition in rice grains (Asaoka et al., 1984, 1985, 1989; Inouchi et al., 2000; Lisle et al., 2000; Umemoto and Terashima, 2002; Cheng et al., 2005; Yamakawa et al., 2007). Heat stress reduced the amylose contents and weakly changed the fine structure of amylopectin (Asaoka et al., 1984; Inouchi et al., 2000), possibly indicating that the abnormal expression of the starch synthesizing enzymes is a key factor causing the chalky grains of rice (Nishi et al., 2001; Tanaka et al., 2004). However, chalky grains without any remarkable change in starch chain distribution were also observed compared to translucent grains which were ripened under both control and HT (control + 3.6°C) conditions (Tsutsui et al., 2013).

Recent remarkable development of the efficient, sensitive, and high-throughput proteomics technology lead us to the next research phase of the grain filling. In the mini review, the current progress of studies on proteome of rice ripening and mature grains is described. Furthermore, the chalking mechanism of rice grain under the HT stress is discussed in terms of grain starch glycome, transcriptome, and proteome.

## RICE GRAIN PROTEOME UNDER HT STRESS

Comprehensive proteomic survey of metabolic enzymes, structural and storage proteins, and allergens in rice grains have been carried out using two-dimensional polyacrylamide gel electrophoresis (2D-PAGE) and gel-free-based shotgun technologies (Koller et al., 2002; Lin et al., 2005; Xu et al., 2008; Lee and Koh, 2011). Lee and Koh (2011) demonstrated that the identification of 4,172 non-redundant proteins with a wide range of molecular weight (5.2–611 kDa) and pI values (pH 2.9–12.6) in developing and mature grains of rice. In the analysis of ontology category enrichment for the 4,172 proteins, 52 categories were enriched, including the carbohydrate metabolic process, transport, localization, lipid metabolic process, and secondary metabolic process. Expression analyses of protein groups associated with different functional categories revealed dynamic changes of metabolisms during rice grain development. It seems that a switch from central carbon metabolism to alcohol fermentation is important for starch synthesis and accumulation in the development process (Xu et al., 2008). Interestingly, however, it was detected that proteins involved in glycolysis, citric acid cycle, lipid metabolism, and proteolysis, and so on, accumulated at higher levels in mature grain than those of developing stages (Lee and Koh, 2011). This observation appears to indicate that the preparation of materials required in germination occurred until the seeds were fully matured and dried.

Information of rice grain proteome in the anthesis and ripening stages under HT stress was limited. The anthesis is the most sensitive stage to HT stress in rice. Jagadish et al. (2010) performed gel-based proteomic analyses of different genotype anthers prepared from rice plants exposed to 6 h of high (38°C) and control (29°C) temperature at anthesis. Both cold (19 kDa) and heat (24 kDa) shock proteins were found significantly up-regulated in highly heat tolerant genotype N22, suggesting that these might contribute to the greater heat tolerance of N22. Lin et al. (2005) have reported that HT stress (35/30°C) during caryopsis development reduced the expression of starch granule-bound starch synthase (Wx), allergen-like proteins, and elongation factor 1β, but enhanced the expression of small heat shock proteins (sHSP), glyceraldehyde-3-phosphate dehydrogenase (GAPDH), and prolamin, in comparison with those in control temperature (30/25°C). Furthermore, they analyzed HT stress response of several different cultivars including high-chalky types, the results showing that sHSP was positively correlated with the appearance of chalky kernels (Lin et al., 2005). Transgenic rice plants overexpressing HSP17.7 (Murakami et al., 2004) and HSFA4d (Yamanouchi et al., 2002) exhibited increase of heat tolerance, however, there was no evaluation during grain filling stage. In recent studies, the accumulation of all classes of storage proteins was increased at early ripening stage under the HT stress, whereas the prolamin accumulation was decreased at maturation and desiccation stages (Lin et al., 2010). On the other hand, Li et al. (2011) descried that pyruvate phosphate dikinase (PPDK) was up-regulated and pullulanase (PUL) was down-regulated during grain filling under the grain chalkiness induced temperature condition, respectively. In wheat grain proteome, HSPs, storage proteins, late embryogenesis abundant proteins, peroxiredoxins, and α-amylase/trypsin inhibitors have shown to be HT-responsive (Skylas et al., 2002; Majoul et al., 2003; Hurkman et al., 2009; Yang et al., 2011). Thus, it must be said that the information concerning the protein expression in developing seeds response to the HT stress is minimum and confusing.

## STARCH GLYCOMIC, TRANSCRIPTOMIC, AND PROTEOMIC ASPECTS OF GRAIN CHALKINESS

Grain chalking caused by HT stress during ripening stage is one of the major issues decreasing the appearance quality of rice grain (Yoshida and Hara, 1977; Tashiro and Wardlaw, 1991a,b). Although the chalky grains of rice were appeared even at the optimum temperature range of grain filling, the HT stress increased the percentage of chalky grains and further extended the chalking area of grain (Tsutsui et al., 2013). Scanning electron microscopy (SEM) studies of the chalky grains have been done by several research groups (Evers and Juliano, 1976; Tashiro and Wardlaw, 1991a; Kim et al., 2000; Lisle et al., 2000; Zakaria et al., 2002). The starch granules in translucent part of chalky grain had similar tight packing and shape to the perfect grain. While, in the opaque part of chalky grains, the starch granules that had a round shape with several small pits were loosely packed (Tsutsui et al., 2013).

It has been demonstrated that the environmental temperature at the ripening stage apparently changes the starch composition in rice grains (Asaoka et al., 1984; Inouchi et al., 2000; Lisle et al., 2000; Umemoto and Terashima, 2002; Cheng et al., 2005; Yamakawa et al., 2007). Interestingly, the enzyme activity of starch branching enzyme IIb (BEIIb) *in vitro* was shown to drop sharply at more than 35°C (Ohdan et al., 2011). The HT stress decreased the amylose contents and the weight ratio of A + short B chains to long B chains of amylopectin in grain (Asaoka et al., 1984; Inouchi et al., 2000), while opposite directions of changes in A- and B-fractions were observed at lower temperatures (Umemoto et al., 1999). Microarray analysis of rice ripening seeds showed that the expression of starch synthesis-related genes including granule-bound starch synthase I**(*GBSSI*), *BEIIb*, ADP-glucose pyrophosphorylase (*AGPS2b, AGPS1, and AGPL2*) and ADP-glucose translocator (*BT1-2*) were partially repressed under HT condition (Yamakawa et al., 2007). It was widely accepted that the *amylose-extender* (*ae*) mutant, that is deficient in *BEIIb* gene, exhibited a severe chalky phenotype of grain. The *ae*-type grains contained amylopectin with largely reduced amount of short chains of degrees of polymerization (DP) 8–12 and enriched in long chains with DP more than 19 (Nishi et al., 2001). The grain chalkiness of *ae* mutant was disappeared by transforming the wild-type *BEIIb* gene (Tanaka et al., 2004), suggesting that the abnormal expression of *BEIIb* is one of factors causing the chalking of grain. However, a recent study showed that the chain-length distributions of starches prepared from the translucent and opaque parts of perfect and chalky grains of Koshihikari cultivar harvested in 2009 (24.4°C) and 2010 (28.0°C) were not distinguishable (Tsutsui et al., 2013). Moreover, Yamakawa et al. (2007) have described that the reduction of amylose content and the increase of long B chains of amylopectin by HT were not correlated to the grain chalkiness. Thus, the relevance of the starch fine structure to the chalkiness of grain under HT stress is still unclear.

The SEM observation indicated that the opaque portion of chalky grain had looser packing of round-shaped starch granules, furthermore, numerous pits were observed on the surface of starch granule from the chalky endosperm (Tashiro and Wardlaw, 1991a; Tsutsui et al., 2013). These observations suggested that, in addition to damage of starch synthesis, premature autolysis of starch induced by HT stress of ripening stage resulted in the abnormal shape of starch granules in the opaque parts of grain. Recently, it was observed that the level of glucose in opaque parts was strikingly high in comparison with the corresponding translucent parts of perfect grains (Tsutsui et al., 2013), possibly indicating that amylolytic enzymes exist and work in the opaque parts of chalky grains. The expression of several α-amylase mRNA species was detected in ripening seeds of rice using a transcriptomic analysis. Noteworthy, the mRNA expression of *Amy1A*, *Amy1C*, *Amy3D*, and *Amy3E* genes, as well as α-amylase activity, was increased under HT stress (Yamakawa et al., 2007; Hakata et al., 2012). In addition, cauliflower mosaic virus 35S promoter-driven overexpression of AmyI-1 (*Amy1A*) and AmyII-4 (*Amy3D*) resulted in grains with decrease weight and chalky appearance even under normal temperature condition (Asatsuma et al., 2006). In marked contrast to the above story, Ishimaru et al. (2009) reported that the α-amylase mRNA was not detected in the central part of endosperm tissue during grain filling, claiming that starch degradation by α-amylase was not the cause of the formation of chalky grain. However, Hakata et al. (2012) have shown that RNAi-mediated suppression of α-amylase genes in ripening seeds resulted in fewer chalky grains under HT conditions, and the extent of the decrease in the ratio of chalky grains was highly correlated to decreases in the gene expression of *Amy1A*, *Amy1C*, *Amy3A*, and *Amy3B*. Furthermore, Tsuyukubo et al. (2010, 2012) have demonstrated by immunoblotting with the specific antibodies that AmyI-1 (Amy1A) and AmyII-4 (Amy3D) proteins existed in the outer layers (100–80% fractions) of rice grain (cv. Koshihikari), while α-glucosidase and AmyII-3 (Amy3E) were mainly detected in the inner layers (90–0% fractions). These experimental results would reveal that activation of amylolytic enzymes by HT is a crucial trigger for grain chalkiness. Local proteomic analyses for determining individual contribution of starch degrading enzymes involving in site-specific localization of chalking remain to be performed.

## FUTURE PERSPECTIVE

**FIGURE 2:**
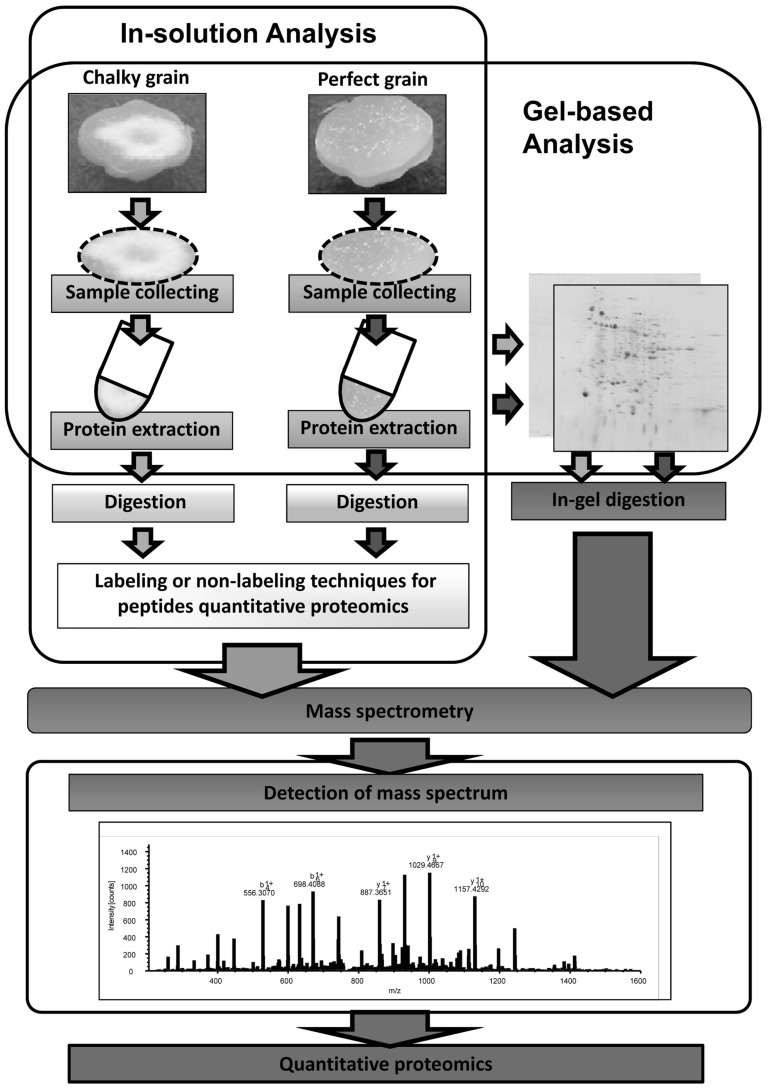
**Local proteomic strategies for understanding the chalking mechanisms of rice grains**.

Global warming is the most serious environmental issue, and the global surface temperature will probably rise a further 1–6°C during the twenty-first century. HT stress in rice ripening periods causes a decrease in not only grain yield but also grain quality. Grain chalking caused by HT stress during ripening stage is one of the major problems in the field of agriculture. Understanding of the mechanisms of grain chalking under HT stress in ripening is extremely important to develop a strategy for reducing the large occurrence of chalky grains in the region to produce good taste and high quality of rice by climate warming. Intensive and precise local proteomic analyses (see **Figure 2**) of HT-stressed developing and mature grains will gain better understanding the grain chalking mechanism(s).

## Conflict of Interest Statement

The authors declare that the research was conducted in the absence of any commercial or financial relationships that could be construed as a potential conflict of interest

## References

[B1] Ainsworth E. A. (2008). Rice production in a changing climate: a meta-analysis of responses to elevated carbon dioxide and elevated ozone concentration. *Glob. Change Biol.* 14 1642–1650

[B2] Ainsworth E. A. Ort D. R. (2010). How do we improve crop production in a warming world? *Plant Physiol.* 154 526–530 20921178 10.1104/pp.110.161349 PMC2949002

[B3] Asaoka M. Okuno K. Fuwa H. (1985). Effect of environmental temperature at the milky stage on amylose content and fine structure of amylopectin of waxy and nonwaxy endosperm starches of rice (*Oryza sativa* L.). *Agric. Biol. Chem.* 49 373–379

[B4] Asaoka M. Okuno K. Hara K. Oba M. Fuwa H. (1989). Effects of environmental temperature at the early developmental stage of seeds on the characteristics of endosperm starches of rice (*Oryza sativa* L.). *Denpun Kagaku* 36 1–8

[B5] Asaoka M. Okuno K. Sugimoto Y. Kawakami J. Fuwa H. (1984). Effect of environmental temperature during development of rice plants on some properties of endosperm starch. *Starch* 36 189–193

[B6] Asatsuma S. Sawada C. Kitajima A. Asakura T. Mitsui T. (2006). α -Amylase affects starch accumulation in the rice grain. *J. Appl. Glycosci.* 53 187–192

[B7] Cheng F. M. Zhong L. J. Wang F. Zhang G. P. (2005). Differences in cooking and eating properties between chalky and translucent parts in rice grains. *Food Chem.* 90 39–46

[B8] Evers A. D. Juliano B. O. (1976). Varietal differences in surface ultrastructure of endosperm cells and starch granules of rice. *Starch* 28 160–166

[B9] Hakata M. Kuroda M. Miyashita T. Yamaguchi T. Kojima M. Sakakibara H. (2012). Suppression of α -amylase genes improves quality of rice grain ripened under high temperature. *Plant Biotechnol. J.* 10 1110–1117 22967050 10.1111/j.1467-7652.2012.00741.x

[B10] Hurkman W. J. Vensel W. H. Tanaka C. K. Whitehand L. Altenbach S. B. (2009). Effect of high temperature on albumin and globulin accumulation in the endosperm proteome of the developing wheat grain. *J. Cereal Sci.* 49 12–23

[B11] Inouchi N. Ando H. Asaoka M. Okuno K. Fuwa H. (2000). The effect of environmental temperature on distribution of unit chains of rice amylopectin. *Starch* 52 8–12

[B12] Ishimaru T. Horigane A. K. Ida M. Iwasawa N. San-oh Y. A. Nakazono M. (2009). Formation of grain chalkiness and changes in water distribution in developing rice caryopses grown under high-temperature stress. *J. Cereal Sci.* 50 166–174

[B13] Jagadish S. V. K. Muthurajan R. Oane R. Wheeler T. R. Heuer S. Bennett J. (2010). Physiological and proteomic approaches to address heat tolerance during anthesis in rice (*Oryza sativa* L.). *J. Exp. Bot.* 61 143–156 19858118 10.1093/jxb/erp289 PMC2791117

[B14] Kim S.-S. Lee S.-E. Kim O.-W. Kim D.-C. (2000). Physicochemical characteristics of chalky kernels and their effects on sensory quality of cooked rice. *Cereal Chem.* 77 376–379

[B15] Koller A. Washburn M. P Lange B. M. Andon N. L. Deciu C. Haynes P. A. (2002). Proteomic survey of metabolic pathways in rice. *Proc. Natl. Acad. Sci. U.S.A.* 99 11969–11974 12163647 10.1073/pnas.172183199 PMC129378

[B16] Lee J. Koh H.-J. (2011). A label-free quantitative shotgun proteomics analysis of rice grain development. *Proteome Sci.* 9 61 10.1186/1477-5956-9-61 PMC3190340 21957990

[B17] Li H. Chen Z. Hu M. Wang Z. Hua H. Yin C. (2011). Different effects of night versus day high temperature on rice quality and accumulation profiling of rice grain proteins during grain filling. *Plant Cell Rep.* 30 1641–1659 21556707 10.1007/s00299-011-1074-2

[B18] Lin C.-J. Li C.-Y. Lin S.-K. Yang F.-H. Huang J.-J. Liu Y.-H. (2010). Influence of high temperature during grain filling on the accumulation of storage proteins and grain quality in rice (*Oryza sativa* L.). *J. Agric. Food Chem.* 58 10545–10552 20839801 10.1021/jf101575j

[B19] Lin S.-K. Chang M.-C. Tsai Y.-G. Lur H.-S. (2005). Proteomic analysis of the expression of proteins related to rice quality during caryopsis development and the effect of high temperature on expression. *Proteomics* 5 2140–2156 15852341 10.1002/pmic.200401105

[B20] Lisle A. J. Martin M. Fitzgerald M. A. (2000). Chalky and translucent rice grains differ in starch composition and structure and cooking properties. *Cereal Chem.* 77 627–632

[B21] Majoul T. Bancel E. Triboï E. Hamida J. B. Branlard G. (2003). Proteomic analysis of the effect of heat stress on hexaploid wheat grain: characterization of heat-responsive proteins from total endosperm. *Proteomics* 3 175–183 12601810 10.1002/pmic.200390026

[B22] Murakami T. Matsuba S. Funatsuki H. Kawaguchi K. Saruyama H. Tanida M. (2004). Over-expression of a small heat shock protein, sHSP17.7, confers both heat tolerance and UV-B resistance to rice plants. *Mol. Breed*. 13 165–175

[B23] Nishi A. Nakamura Y. Tanaka N. Satoh H. (2001). Biochemical and genetic analysis of the effects of amylose-extender mutation in rice endosperm. *Plant Physiol.* 127 459–472 11598221 PMC125082

[B24] Ohdan T. Sawada T. Nakamura Y. (2011). Effects of temperature on starch branching enzyme properties of rice. *J. Appl. Glycosci.* 58 19–26

[B25] Peng S. Huang J. Sheehy J. E. Laza R. C. Visperas R. M. Zhong X. (2004). Rice yields decline with higher night temperature from global warming. *Proc. Natl. Acad. Sci. U.S.A.* 101 9971–9975 15226500 10.1073/pnas.0403720101 PMC454199

[B26] Skylas D. J. Cordwell S. J. Hains P. G. Larsen M. R. Basseal D. J. Walsh B. J. (2002). Heat shock of wheat during grain filling: Proteins associated with heat-tolerance. *J. Cereal Sci.* 35 175–188

[B27] Tanaka N. Fujita N. Nishi A. Satoh H. Hosaka Y. Ugaki M. (2004). The structure of starch can be manipulated by changing the expression levels of starch branching enzyme IIb in rice endosperm. *Plant Biotechnol. J.* 2 507–516 17147623 10.1111/j.1467-7652.2004.00097.x

[B28] Tashiro T. Wardlaw I. F. (1991a). The effect of high temperature on the accumulation of dry matter, carbon and nitrogen in the kernel of rice. *Aust. J. Plant Physiol.* 18 259–265

[B29] Tashiro T. Wardlaw I. F. (1991b). The effect of high temperature on kernel dimensions and the type and occurrence of kernel damage in rice. *Aust. J. Agric. Res.* 42 485–496

[B30] Tsutsui K. Kaneko K. Hanashiro I. Nishinari K. Mitsui T. (2013). Characteristics of opaque and translucent parts of high temperature stressed grains of rice. *J. Appl. Glycosci.* 10.5458/jag.jag.JAG-2012 014

[B31] Tsuyukubo M. Ookura T. Mabashi Y. Kasai M. (2010). Different distributions of α -glucosidases and amylases in milling fractions of rice grains. *Food Sci. Technol. Res.* 16 523–530

[B32] Tsuyukubo M. Ookura T. Tsukui S. Mitsui T. Kasai M. (2012). Elution behavior analysis of starch degrading enzymes during rice cooking with specific antibodies. *Food Sci. Technol. Res.* 18 659–666

[B33] Umemoto T. Nakamura Y. Satoh H. Terashima K. (1999). Differences in amylopectin structure between two rice varieties in relation to the effects of temperature during grain-filling. *Starch/Stärke* 51 58–62

[B34] Umemoto T. Terashima K. (2002). activity of granule-bound starch synthase is an important determinant of amylose content in rice endosperm. *Funct. Plant Biol.* 29 1121–1124 10.1071/PP01145 32689564

[B35] Xu S. B. Li T. Deng Z. Y. Chong K. Xue Y. Wang T. (2008). Dynamic proteomic analysis reveals a switch between central carbon metabolism and alcoholic fermentation in rice filling grains. *Plant Physiol.* 148 908–925 18753281 10.1104/pp.108.125633 PMC2556828

[B36] Yamakawa H. Hirose T. Kuroda M. Yamaguchi T. (2007). Comprehensive expression profiling of rice grain filling-related genes under high temperature using DNA microarray. *Plant Physiol.* 144 258–277 17384160 10.1104/pp.107.098665 PMC1913800

[B37] Yamanouchi U. Yano M. Lin H. Ashikari M. Yamada K. (2002). A rice spotted leaf gene, Spl7, encodes a heat stress transcription factor protein. *Proc. Natl. Acad. Sci. U.S.A.* 94 7530–7535 12032317 10.1073/pnas.112209199 PMC124274

[B38] Yang F. Jørgensen A. D. Li H. Søndergaard I. Finnie C. Svensson B. (2011). Implications of high-temperature events and water deficits on protein profiles in wheat (*Triticum aestivum* L. cv. Vinjett) grain. *Proteomics* 11 1684–1695 21433286 10.1002/pmic.201000654

[B39] Yoshida S. Hara T. (1977). Effects of air temperature and light on grain filling of an indica and a japonica rice (*Oryza sativa* L.) under controlled environmental conditions. *Soil Sci. Plant Nutr.* 23 93–107

[B40] Zakaria S. Matsuda T. Tajima S. Nitta Y. (2002). Effect of high temperature at ripening stage on the reserve accumulation in seed in some rice cultivars. *Plant Prod. Sci.* 5 160–168

